# Reversal of carbonate-silicate cation exchange in cold slabs in Earth’s lower mantle

**DOI:** 10.1038/s41467-021-21761-9

**Published:** 2021-03-17

**Authors:** Mingda Lv, Susannah M. Dorfman, James Badro, Stephan Borensztajn, Eran Greenberg, Vitali B. Prakapenka

**Affiliations:** 1 Department of Earth and Environmental Sciences, Michigan State University, East Lansing, MI USA; 2 Université de Paris, Institut de physique du globe de Paris, CNRS, Paris, France; 3 Center for Advanced Radiation Sources, University of Chicago, Chicago, IL USA; 4 Present Address: Applied Physics Department, Soreq Nuclear Research Center (NRC), Yavne, 81800 Israel

**Keywords:** Solid Earth sciences, Geochemistry, Mineralogy

## Abstract

The stable forms of carbon in Earth’s deep interior control storage and fluxes of carbon through the planet over geologic time, impacting the surface climate as well as carrying records of geologic processes in the form of diamond inclusions. However, current estimates of the distribution of carbon in Earth’s mantle are uncertain, due in part to limited understanding of the fate of carbonates through subduction, the main mechanism that transports carbon from Earth’s surface to its interior. Oxidized carbon carried by subduction has been found to reside in MgCO_3_ throughout much of the mantle. Experiments in this study demonstrate that at deep mantle conditions MgCO_3_ reacts with silicates to form CaCO_3_. In combination with previous work indicating that CaCO_3_ is more stable than MgCO_3_ under reducing conditions of Earth’s lowermost mantle, these observations allow us to predict that the signature of surface carbon reaching Earth’s lowermost mantle may include CaCO_3_.

## Abstract (web summary)

CaCO_3_ is more stable than MgCO_3_ under conditions of Earth’s lowermost mantle, with implications for deep mantle chemistry.

## Introduction

Carbon is not the only key to life and Earth’s habitability but also traces and modifies geological processes of subduction, partial melting, degassing, and metasomatism, providing valuable insights into Earth’s evolution^1^. Over the history of the planet, carbon transport between surface and deep reservoirs has impacted the atmospheric, oceanic, and crustal CO_2_ budgets in tandem with the composition and redox state of the Earth’s mantle^2,3^. Carbon is transported from Earth’s surface to its interior mainly as carbonate minerals in subduction zones and is returned in carbon-bearing gas/fluid through volcanic degassing^2,3^. These processes leave signatures in the mantle, including depletion of incompatible elements^4,5^, diamond formation (and inclusions)^6,7^, and isotopic abundances^8,9^. Carbon flux via subduction to the deep mantle remains uncertain, with estimated magnitudes ranging from 0.0001 to 52 megatons/year^3,10^. The wide range of these estimates is due in part to limited understanding of the physical and chemical responses of carbonates to mantle pressures, temperatures, and compositional environments.

The dominant carbonates carried into the mantle by subducting slabs, dolomite CaMg(CO_3_)_2_, magnesite MgCO_3,_ and calcite CaCO_3_^11^, undergo changes in crystal structure or state and chemical reactions at depth. Carbonates are likely to be retained as solid minerals in subducting ocean crust until/unless the solidus of carbonated peridotite^12,13^ or eclogite^14,15^ intersects with mantle geotherms, initiating melting. These slab-derived carbonatite melts will segregate to the overlying mantle due to low viscosity and density^16^, or be reduced to diamonds at depths greater than ~250 km via redox freezing^7,15,17^. However, carbonates are present in the mantle transition zone and possibly lower-mantle depths in some regions, based on direct evidence provided by carbonate minerals found in deep-sourced diamond inclusions^18,19^. Additional evidence from thermodynamic modeling of devolatilization of carbonate-bearing subducting slab^20,21^ and melting experiments on carbonates in the MgCO_3_–CaCO_3_ system up to 80 GPa^22^ supports the preservation of solid carbonates along low-temperature geotherms in subducting slabs in the lower mantle. However, the temperature is not the only control on the fate of subducted carbonates: carbonates may also interact chemically with the major phases of the ambient mantle or basalt-rich subducted crust. In these compositions in the lower mantle, the silicates potentially reacting with carbonates are bridgmanite (bdg), post-perovskite (pPv), and Ca-perovskite (Ca-Pv).

The presence of the end-member carbonates, MgCO_3_ and CaCO_3_ (note that (Mg,Ca)(CO_3_)_2_ dolomite breaks down to these end-members above 5 GPa and 1200 K^23^), together with lower-mantle silicates depends on the thermodynamics and kinetics of the carbonate–silicate exchange reaction:

1 $${\mathrm{CaCO}}_{3}+{\mathrm{MgSiO}}_{3}\rightarrow {\mathrm{MgCO}}_{3}+{\mathrm{CaSiO}}_{3}$$

Previous experiments^24,25^ indicate that CaCO_3_ reacts with silicates to form MgCO_3_ via the forward reaction up to 80 GPa and 2300 K, i.e., at least to the mid-lower mantle. Theoretical studies further predict that MgCO_3_ + CaSiO_3_ are enthalpically favored over CaCO_3_ + MgSiO_3_ throughout the lower mantle pressure and temperature regime^26–30^. However, although many studies have addressed the stability of individual carbonates up to higher pressures^31–33^, no experiments examined the carbonate–silicate cation exchange reaction up to core–mantle boundary conditions.

In this work, to assess the stability of MgCO_3_ and CaCO_3_ coexisting with lower-mantle silicates, we conduct a series of experiments on the carbonate–silicate reaction along the lower-mantle geotherm. Thin disks of carbonates and silicates were loaded together in laser-heated diamond-anvil cells (LHDAC, Supplementary Table 1, see “Methods” for details). Laser heating at 1600–2800 K and 33–137 GPa was applied for 10–400 min. Run products were examined by in situ synchrotron X-ray diffraction (XRD) and ex situ energy-dispersive X-ray spectroscopy (EDX) analysis with a scanning transmission electron microscope (STEM, see “Methods” for details).

## Results

### Calcium carbonate reaction to form magnesium carbonate

Experiments assessed thermodynamic stability by using as reactants either (Mg,Ca)CO_3_ + (Mg,Fe)SiO_3_ (reactants for the forward reaction, hereafter referred to CaC-to-MgC) and (Mg,Fe)CO_3_ + CaSiO_3_ (reactants for the reverse reaction, hereafter referred to MgC-to-CaC). For reaction CaC-to-MgC, the criterion for determining whether the reaction takes place is the presence of newly synthesized CaSiO_3_-perovskite in the run product. For reaction MgC-to-CaC, newly synthesized MgSiO_3_ and CaCO_3_ indicate the reaction is favorable. The silicate reaction products are easier to observe through diffraction than carbonates due to higher diffraction intensity.

**Fig. 1:**
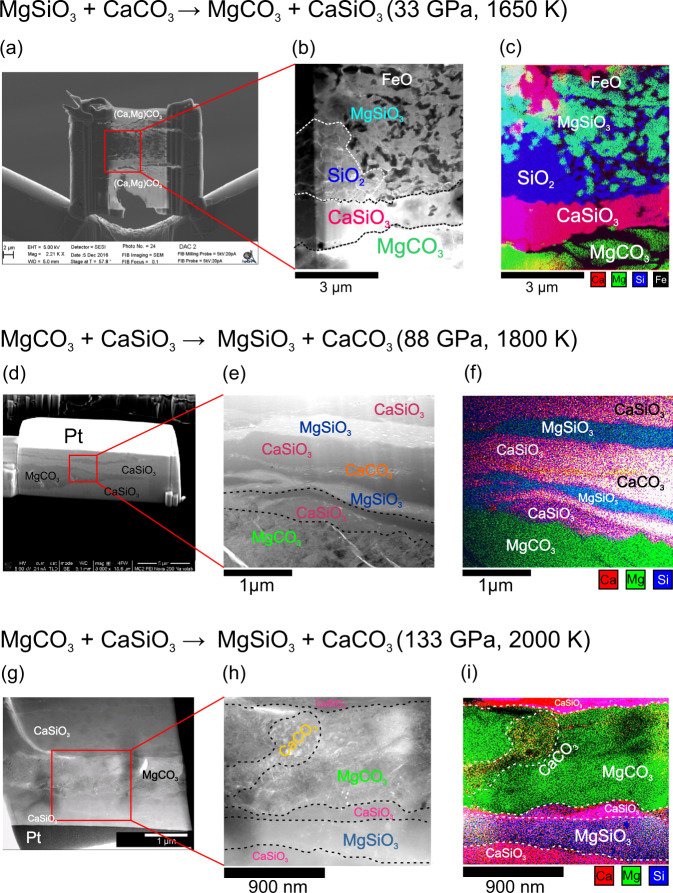
Electron microscopic characterizations of recovered samples. Images of selected recovered sample cross-sections obtained using backscattered scanning electron microscopy (**a**, **d**, **g**), scanning transmitted electron microscopy (**b**, **e**, **h**), and energy-dispersive X-ray mapping (**c**, **f**, **i**) of the cross-section show the silicate layer sandwiched by two carbonate layers, with the reaction region along the contacting interface. **a**–**c** Ex situ analysis of sample quenched from 33 GPa and 1650 K heated for 15 min (run #1) demonstrates reaction CaC-to-MgC: CaSiO_3_ is not present in starting materials but is indicated in EDX map by colocation of Ca and Si, shown in magenta. **d**–**f** Ex situ analysis of sample quenched from 88 GPa and 1800 K heated for 150 min (run #9) demonstrates reaction MgC-to-CaC: MgSiO_3_ is not present in starting materials but is indicated in EDX map by colocation of Mg and Si, shown in blue-green. CaCO_3_ also appears as a red (Ca, but no Si) ribbon within CaSiO_3_ starting material. **g**–**i** Ex situ analysis of sample quenched from 133 GPa and 2000 K heated for 400 min (run #10) demonstrates reaction MgC-to-CaC: MgSiO_3_ appears as Ca-depleted, Si-rich region (blue or blue-green) adjacent to CaSiO_3_ starting material (magenta).

Experiments with CaC-to-MgC reactants indicate the forward reaction takes place in runs conducted below 83 GPa (runs #1–4), as determined via both EDX and XRD. For example, ex situ electron microscopic analysis of the sample recovered from 33 GPa and 1650 K (run #1) (Fig. 1a–c) reveals a ~1-μm-thick layer of CaSiO_3_ between the silicate layer and the carbonate layer, coexisting with SiO_2_, FeO, MgSiO_3_, and MgCO_3_. These observations are consistent with in situ XRD patterns of run products after heating (Supplementary Figs. 3b and  5a), which exhibit several new sharp peaks compared to the pattern before heating (Supplementary Fig. 3a). The diffraction pattern of run products is consistent with the presence of Ca-Pv, magnesite, bdg, wüstite, stishovite, and monoclinic dolomite III (previously observed at pressure above 36 GPa^34^). Ca-Pv can be observed in the run products of CaC-to-MgC up to 83 GPa (Supplementary Figs. 3c and  4a, b), in agreement with previous experimental observations^24,25^.

At higher pressures from 91 to 137 GPa, however, we observe no evidence of carbonate–silicate exchange reaction in experiments with CaC-to-MgC reactants. Ca-Pv is not identified in the run products (runs #5-7) through either in situ (Supplementary Figs. 3d, e,  4c, d, and 5b) or ex situ analysis. New, sharp peaks from bdg and pPv can be observed in situ in XRD patterns (Supplementary Fig. 3d, e), indicating the sample was sufficiently heated to transform starting materials to high-pressure silicate structures, but no carbonate–silicate exchange reaction occurs. Two hypotheses can explain these observations: (1) in contrast to theoretical predictions that the reversal of the carbonate-exchange reaction takes place at higher pressures and lower temperatures^26–30^, CaCO_3_ + MgSiO_3_ become more favorable than MgCO_3_ + CaSiO_3_ from 91 to 137 GPa and 2100 to 2800 K; (2) the reaction CaC-to-MgC is hindered by reaction kinetics, and metastable starting materials are observed.

### Magnesium carbonate reaction to form calcium carbonate

In order to resolve the thermodynamically stable phase assemblage, three separate sets of experiments on the backward reaction (MgC-to-CaC, runs #8–11) were conducted at 35–133 GPa and 1800–2000 K. Elemental mapping of the run products of experiments at 88 GPa (run #10, Fig. 1d–f) and 133 GPa (run #11, Fig. 1g–i) indicates that MgSiO_3_ layers formed along with the carbonate–silicate interface, and newly formed CaCO_3_ can be observed as well. At 35 GPa, neither EDX nor XRD shows MgSiO_3_ formed from MgC-to-CaC reactants (run #8, Supplementary Fig. 9). Observations of the reversal of the reaction confirm that MgCO_3_ is unstable and reacts with CaSiO_3_ producing CaCO_3_ and MgSiO_3_ at pressures higher than 88 GPa along a lower-mantle geotherm.

**Fig. 2:**
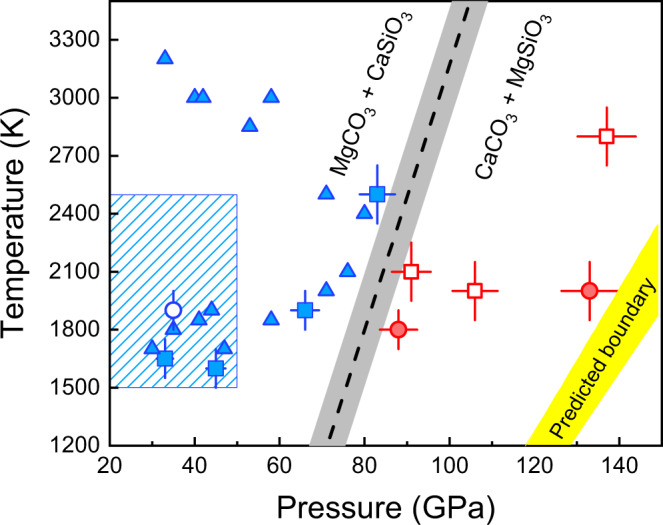
Phase diagram for the relative stability of the MgCO_3_ + CaSiO_3_ assemblage versus CaCO_3_ + MgSiO_3_. The boundary sketched as a black dashed line with gray shadow as uncertainty inferred is based on experimental observations of carbonate–silicate exchange reactions CaC-to-MgC and MgC-to-CaC. Squares represent observations from this work starting with (Ca,Mg)CO_3_ and (Mg,Fe)SiO_3_, looking for newly synthesized CaSiO_3_ to indicate the CaC-to-MgC reaction takes place. Circle symbols represent observations from this work of experiments starting with (Mg,Fe)CO_3_ + CaSiO_3_, looking for identification of newly synthesized MgSiO_3_ to indicate the MgC-to-CaC reaction takes place. Open symbols indicate nonreaction and filled for confirmed reaction, and blue and red colors correspond to the inferred stable phase assemblage based on reaction products. Triangles indicate the *P*–*T* conditions for CaC-to-MgC taking place reported by Seto et al.^25^, and blue-shaded region indicates approximate conditions of four experiments conducted by Biellmann et al.^24^ using indirect methods for pressure and temperature calibration, which all produced the run products MgCO_3_ + CaSiO_3_. The error bars indicate uncertainties of pressure and temperature measurements (see “Methods” for details). The boundaries proposed by previous theoretical predictions are illustrated by yellow-shaded region^28–30^.

Our results agree with previous experimental constraints (Fig. 2) below 80 GPa showing: dolomite is unstable relative to CaCO_3_ and MgCO_3_ at lower-mantle conditions^23–25,35^; neither CaO nor MgO are observed in run products, indicating no decomposition of CaCO_3_ and MgCO_3_ into oxides plus CO_2_^26,27,30^; MgCO_3_ is more favorable in the lower mantle than CaCO_3_ up to ~80 GPa due to the CaC-to-MgC reaction^24,25^. Since similar previous studies were limited to pressures below 80 GPa, they did not observe the reversal reaction (MgC-to-CaC). Combining our new results with previous results^24,25^ and theoretical predictions indicating a positive Clapeyron slope for this reaction^28–30^, we suggest a reaction boundary above 80 GPa with a positive slope (black dashed line in Fig. 2). We note that the experimental data allow for significant uncertainty in this boundary, but are inconsistent with theoretical predictions^28–30^ (yellow region, Fig. 2). This discrepancy may have been produced by theoretical approximations at higher temperatures. If density functional perturbation theory and quasi-harmonic approximation have misestimated the volumes of the carbonate phases expected to be stable at ~80 GPa and higher pressures, this could lead to the systematic overestimation of Gibbs free energy of CaCO_3_ + MgSiO_3_ relative to MgCO_3_ + CaSiO_3_ at higher temperatures.

## Discussion

**Fig. 3:**
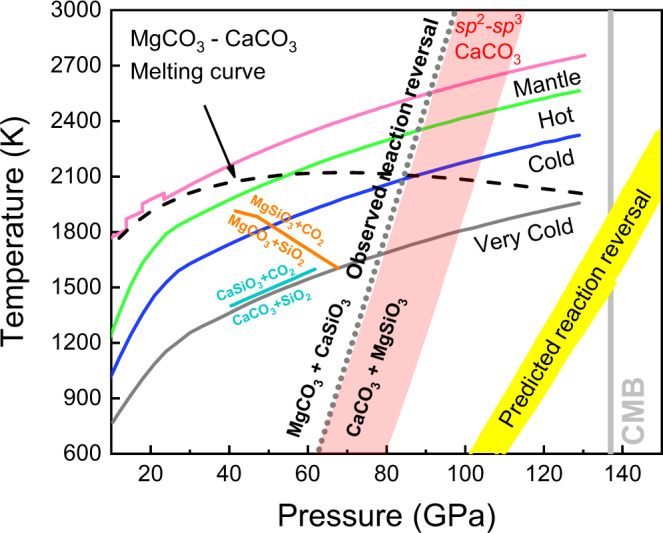
Pressure–temperature diagram of reactions between carbonate, silicates, and silica in the subducted oceanic crust to the lower mantle. The gray dotted line indicates the reversal boundary of the carbonate–silicate exchange reaction proposed by this study, whereas previous theoretical predictions are illustrated by yellow-shaded region^28–30^. The cyan and orange lines indicate the decarbonation reactions of CaCO_3_ + SiO_2_^40^ and MgCO_3_ + SiO_2_^41^, respectively. The black dashed line shows the melting curve of MgCO_3_–CaCO_3_ system constrained by Thomson et al.^22^. Four typical mantle geotherms are modified from Maeda et al.^36^. The red-shaded region indicates the transition boundary of CaCO_3_ from *sp*^2^ to *sp*^3^ structure predicted by density functional theory computations^29,38^.

The pressure/temperature conditions of the reversal reaction as constrained by these experiments are similar to those of polymorphic phase transitions associated with *sp*^2^–*sp*^3^ bonding changes in both MgCO_3_ and CaCO_3_, which suggests these transitions are related to the stabilization of a CaCO_3_ + MgSiO_3_ assemblage. The transition from *sp*^2^- to *sp*^3^ bonds in MgCO_3_ has been identified at ~80 GPa with the stabilization of the *C*2/*m* structure^32,33,36^, and the resulting densification of MgCO_3_ supports the forward reaction to MgCO_3_ + CaSiO_3_. The transition in CaCO_3_ from *sp*^2^- to *sp*^3^ bonds in the *P*2_1_/*c*-h structure was experimentally observed at ~105 GPa and 2000 K^37^. Computational studies predicted this boundary at ~70^29^ and ~100 GPa^38^ at mantle-relevant temperatures (red-shaded region in Fig. 3). While an earlier study that did not include the *sp*^3^ CaCO_3_-*P*2_1_/*c*-h structure predicted a crossover in silicate–carbonate-exchange reaction at 135 GPa and 0 K^26^, a later study that predicted the *sp*^3^ CaCO_3_-*P*2_1_/*c*-h structure found a silicate–carbonate reaction reversal at 84 GPa and 0 K^30^. This would correspond to *sp*^2^–*sp*^3^ crossover and stabilization of CaCO_3_ + MgSiO_3_ in the mid-lower mantle.

**Fig. 4:**
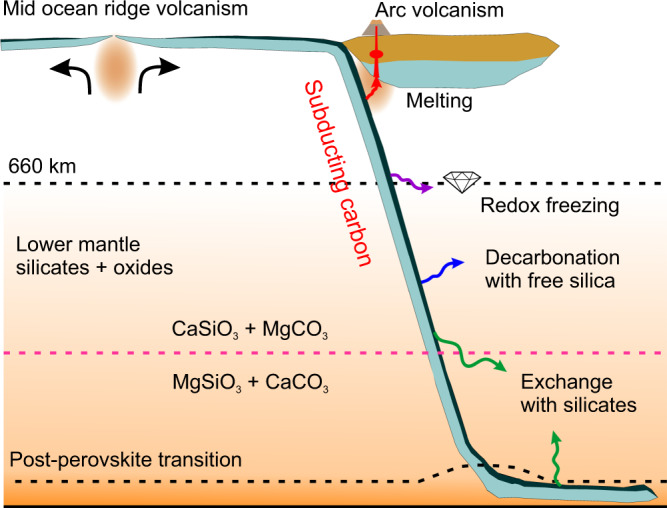
Schematic illustration of the fate of carbonates in the oceanic crust (dark blue) subducted to the lower mantle. Through subduction, the carbonates may undergo melting (red arrow), redox freezing with metallic iron (purple arrow), decarbonation reaction with free silica (blue arrow), and exchange reaction with lower-mantle silicates (green arrow). Based on the observation of reversal of the carbonate–silicate cation exchange reaction at conditions relevant to cold subducted slabs at mid-lower-mantle depths, CaCO_3_ is the potential stable phase that hosts oxidized carbon in the lowermost mantle.

Whether a crossover in the carbonate–silicate exchange reaction takes place in the deep Earth depends on whether carbonates are preserved in Earth’s lower mantle to at least 1800-km depth. Previous studies have identified barriers to carbon subduction and stability in the lower mantle, particularly melting^15,39^ and reduction^40–42^. If carried in cold subducting slabs, MgCO_3_ and CaCO_3_ may avoid melting as their melting temperatures^22^ are higher than some predicted cold slab geotherms^36^. Any solid carbonate in the mantle will be in contact and may equilibrate with silicates in all mantle environments and with free silica in basalt-rich compositions. MgCO_3_ and CaCO_3_ have been observed in experiments^40–42^ to undergo decarbonation reactions with free silica over a pressure range of ~40 to 60 GPa. However, the Clapeyron slope of CaCO_3_ + SiO_2_ → CaSiO_3_ + CO_2_ is positive and takes place at pressure/temperature conditions warmer than the coolest slab geotherms^40^. Observations that MgCO_3_ is less thermally stable than CaCO_3_ support the survival of CaCO_3_ rather than MgCO_3_ along a cold subducted slab geotherm to the lowermost mantle^36,41^ (Fig. 3). In this study, we report a reversal in the Mg–Ca silicate–carbonate cation exchange reaction at ~90 GPa, making MgCO_3_ + CaSiO_3_ favorable in the upper part of the lower mantle, while CaCO_3_ + MgSiO_3_ is preferred in the lower part of the lower mantle (Fig. 3). However, the question of whether any carbonate persists to these depths in the coldest subducting slabs remains unresolved. If MgCO_3_ remains present in cold slabs, and the reaction CaC-to-MgC proceeds throughout most of the mantle eliminating CaCO_3_, the reversal MgC-to-CaC reaction may transform MgCO_3_ back to CaCO_3_ in the lowermost mantle (Fig. 4). CaCO_3_ could thus be found in the lowermost mantle coexisting with silicates and reduced iron.

The reduced nature of the Earth’s mantle, with oxygen fugacity inferred to be near the iron–wüstite buffer in the transition zone and greater depths^43^, stabilizes diamond or Fe carbide as long-term hosts of carbon, owing to their chemical refractoriness and dynamic immobility^44^. Similarly, our experimental observations support CaCO_3_ as a refractory, stable host for oxidized carbon in the middle to the lowermost mantle, in particular, the high-pressure polymorph of CaCO_3_ (CaCO_3_-*P*2_1_/*c*-h) with tetrahedral bonds^37^. Experimental observations also suggest that CaCO_3_ is more resistant to redox breakdown reaction with iron under reduced conditions than MgCO_3_^35^. In addition, due to the cation exchange between carbonate and silicate, the relative stability of MgCO_3_ or CaCO_3_ will change in the lowermost mantle, and depending on conditions one of these phases may buffer the redox state of the mantle through an influx of oxidized carbon in the form of solid carbonate^45^.

The Mg–Ca silicate–carbonate-exchange reactions along with subduction pressure–temperature (*P*–*T*) conditions may impact observable signatures of Mg and Ca isotopes in mantle silicates under certain special conditions, or in carbonate inclusions in diamonds. Subducting carbonates carry low-δ^44/40^Ca and low-δ^26^Mg signatures relative to the heavier mantle ratios, but although previous studies have observed heterogeneity in the Ca and Mg isotope signatures in basalts and mantle peridotites, these studies determined that lighter ratios cannot be simply interpreted as evidence of recycled marine carbonates^46,47^. The Mg–Ca silicate–carbonate-exchange reactions along with subduction *P*–*T* conditions may contribute to these variable Mg and Ca isotopic compositions. The reaction CaC-to-MgC in the transition zone and upper part of the lower mantle would transfer light Ca isotopes from subducted CaCO_3_ to CaSiO_3_ (Ca-Pv) (Supplementary Note 1 and Supplementary Fig. 12). Isotopically light Ca-Pv can then be trapped in diamond inclusions and return to the surface^48^, while the Ca isotopic signature of upwelling rocks would remain variable, as it undergoes continuous fractionation within peridotitic mantle lithologies^46,49–51^. The modification of carbonate–silicate phase equilibria observed in this study provides a new process that could alter Mg and Ca isotopic composition in such lithologies (Supplementary Note 1 and Supplementary Fig. 12). While the isotope signature of MgSiO_3_ produced by reaction MgC-to-CaC would not be observable due to the small masses involved relative to the vast lower-mantle reservoir of MgSiO_3_, any CaCO_3_ produced in the deep lower mantle by this reaction would carry a heavier deep mantle δ^44/40^Ca signature that would distinguish it from surface-derived carbonate. If preserved in diamond inclusions and returned to the surface, heavy CaCO_3_ could be used to trace the presence of oxidized carbon in the lowermost mantle. The potential of CaCO_3_ to be a signature of an ultradeep carbon cycle reaching the core–mantle-boundary region may help to reveal other mysteries of the deep mantle, such as heat budget related to radioactive elements stored in Ca-bearing silicates^52^, and compositions of heterogeneities that may record Earth’s early history^48,53^.

## Methods

### Starting materials

To investigate phase equilibria in the carbonate–silicate system in Earth’s lower mantle and control for effects of reaction kinetics, both CaC-to-MgC and MgC-to-CaC experiments were carried out in symmetric diamond-anvil cells (DAC) with flat-top double-sided laser heating^54^. For CaC-to-MgC, natural dolomite with homogeneous composition of (Mg_0.38_Ca_0.59_Fe_0.03_)CO_3_ was used as a carbonate reactant, the composition, and structure of which has been characterized by X-ray fluorescence spectroscopy and X-ray diffraction, respectively^35^. Fe-bearing enstatite synthesized at École Polytechnique Fédérale de Lausanne with a composition of (Mg_0.5_Fe_0.5_)SiO_3_ was used as a silicate reactant^55^. For MgC-to-CaC, natural ferromagnesite (sample from Princeton University) was used as a carbonate reactant, with composition determined to be (Mg_0.87_Fe_0.13_)CO_3_ by wavelength dispersive X-ray spectroscopy in a Cameca SX100 Electron Probe Microanalyzer at the University of Michigan. Pure calcium silicate (CaSiO_3_, Alfa Aesar) was used as a silicate reactant. The chief advantages to the abovementioned starting compositions are that recognition of a carbonate–silicate exchange reaction only requires identification of the presence of newly synthesized silicates in quenched run products, i.e., Ca-perovskite (Ca-Pv) in CaC-to-MgC and bridgmanite (bdg) in MgC-to-CaC; and Fe-bearing enstatite and ferromagnesite can serve as laser absorber during the forward CaC-to-MgC and reversal MgC-to-CaC experiments, respectively.

### LHDAC experiments

The dolomite, enstatite, and calcium silicate samples were separately ground under acetone in an agate mortar for ~2 h each to achieve homogenous, finely powdered samples with grain size typically less than ~2 µm. A single ferromagnesite crystal was double-side polished to ~10-micron thickness. All starting materials were dried in an oven at 120 °C overnight before loading, and the powder samples were subsequently pressed in a DAC to form thin foils approximately ~8–10-µm thick. The enstatite foils and ferromagnesite crystals were sandwiched between iron-free dolomite and calcium silicate, respectively, serving as thermal insulators in symmetric DACs for CaC-to-MgC and MgC-to-CaC (Supplementary Figs. 1 and2). No other pressure standard or medium was loaded to prevent reactions with other components and contamination of the chemical system. The sample sandwiches were loaded in sample chambers with diameters approximately halves of the anvil culet sizes drilled into Re gaskets pre-indented to a thickness of ∼30 μm, by using the laser drilling system at HPCAT (Sector 16) of the Advanced Photon Source (APS), Argonne National Laboratory (ANL)^56^. Diamond anvils with flat culets of 300 μm were used for experiments under 60 GPa, beveled culets of 150/300 μm for experiments under 100 GPa, and beveled culets of 75/300 μm for experiments up to 140 GPa.

Before laser heating, each sample was compressed to the target pressure at 300 K, and after heating each sample was quenched to ambient pressure at 300 K to limit and preserve reactions at target conditions. Pressures were determined from the Raman shift of the singlet peak of the diamond anvil at the culet surface^57^, and post-heating pressures were typically within 3% of the pre-heating pressure. Thermal pressure during heating may be estimated to be ~10% GPa higher than the pre-heating pressure at the modest temperatures^58,59^. High-temperature conditions were achieved by using a double-sided ytterbium fiber laser heating system at beamline 13-ID-D (GeoSoilEnviroCars) of APS, ANL^54^, with two 1.064 μm laser beams focused down to a flat-top spot with a diameter of 10–12 μm on both sides of the sample. Temperatures of the heated samples were determined by fitting the measured thermal radiation spectra using the Planck radiation function under the graybody approximation^54^. The temperature reported in Supplementary Table 1 is the temporal average of multiple temperature measurements over the heating duration. Temperature fluctuations over this timescale were less than the specified uncertainty, which is derived from a standard deviation of temperature measurements from both sides of the laser-heated sample (typically ± 100 K below 2000 K and ±150 K above 2000 K) (Supplementary Figs. 10 and  11). Experiments were held at temperatures between 1600 and 2800 K for ~30 min in CaC-to-MgC experiments and up to 400 min in MgC-to-CaC experiments.

### In situ XRD

Phases synthesized at high *P*/*T* and achievement of chemical steady-state were determined by in situ angle-dispersive X-ray diffraction (XRD) measurements performed before, during, and after heating at beamline 13-ID-D (GeoSoilEnviroCars) of APS, ANL. The incident X-ray beam was focused down to less than 3 × 4 μm^2^ with a monochromatic wavelength *λ* = 0.3344 Å. Diffracted X-rays were recorded using a MAR 165 detector or Pilatus 1 M CdTe pixel array detector. NIST standard LaB_6_ was used to calibrate the detector distance, tilt angle, and rotation angle of the image plane relative to the incident X-ray beam. Exposure times were typically 30 s. The XRD patterns were integrated to produce 2*θ* plots using the software DIOPTAS^60^.

### Ex situ EDX

After complete pressure release, each sample was recovered from the LHDAC, and then sectioned along the compression axis through the laser-heated spot and over the entire thickness of the DAC sample (~5–20 μm), using a focused ion beam (FIB) coupled with a field-emission scanning electron microscope (FE-SEM) at IPGP (Paris, France) or the Michigan Center for Materials Characterization at the University of Michigan (Ann Arbor, USA). A ~30-nm-thick Au layer was coated on each sample to reduce charging in the scanning electron microscope, and a 2-μm-thick Pt layer was deposited across the center of each heated spot to protect the sample from damage by the Ga^+^ ion beam. Thin sections of each heated spot were extracted and polished to electron transparency (∼100-nm thickness).

Textural and chemical characterization of recovered samples was performed with scanning transmission electron microscopy (STEM) and energy-dispersive X-ray spectroscopy (EDX) in a JEOL 2200FS field-emission TEM (Center for Advanced Microscopy, MSU), operated at 200 kV to image the sample in brightfield. EDX maps were scanned over 512 × 384 pixel areas with a pixel dwell time of 50 μs. Typical count rates were ~2000 counts per second. Chemical mapping rather than point measurement approach prevents migration of elements due to damage by the electron beam. Uncertainties in compositions were determined from standard deviations of EDX measurements obtained from selected regions within multiple grains.

## Supplementary information

Supplementary Information

Peer Review File

## Data Availability

Additional diffraction and spectroscopy data and metadata are available in the Supplementary Information and from the corresponding authors upon request.

## References

[1] Hazen RM, Schiffries CM (2013). Why deep carbon?. Rev. Mineral. Geochem..

[2] Plank T, Manning CE (2019). Subducting carbon. Nature.

[3] Kelemen PB, Manning CE (2015). Reevaluating carbon fluxes in subduction zones, what goes down, mostly comes up. Proc. Natl Acad. Sci. USA.

[4] Stachel T (2004). The trace element composition of silicate inclusions in diamonds: a review. Lithos.

[5] Thomson AR (2016). Trace element composition of silicate inclusions in sub-lithospheric diamonds from the Juina-5 kimberlite: evidence for diamond growth from slab melts. Lithos.

[6] Palyanov YN (2013). Mantle-slab interaction and redox mechanism of diamond formation. Proc. Natl Acad. Sci. USA.

[7] Rohrbach A, Schmidt MW (2011). Redox freezing and melting in the Earth’s deep mantle resulting from carbon-iron redox coupling. Nature.

[8] Cartigny P, Palot M, Thomassot E, Harris JW (2014). Diamond formation: a stable isotope perspective. Annu. Rev. Earth Planet. Sci..

[9] Teng F-Z (2017). Magnesium isotope geochemistry. Rev. Mineral. Geochem..

[10] Dasgupta R, Hirschmann MM (2010). The deep carbon cycle and melting in Earth’s interior. Earth Planet Sci. Lett..

[11] Poli S, Schmidt MW (2002). Petrology of subducted slabs. Annu. Rev. Earth Planet. Sci..

[12] Ghosh S, Litasov K, Ohtani E (2014). Phase relations and melting of carbonated peridotite between 10 and 20 GPa: a proxy for alkali- and CO_2_-rich silicate melts in the deep mantle. Contrib. Miner. Pet..

[13] Dasgupta R, Hirschmann MM (2006). Melting in the Earth’s deep upper mantle caused by carbon dioxide. Nature.

[14] Kiseeva ES (2012). An experimental study of carbonated eclogite at 3.5-5.5 GPa-implications for silicate and carbonate metasomatism in the cratonic mantle. J. Petrol..

[15] Thomson AR, Walter MJ, Kohn SC, Brooker RA (2016). Slab melting as a barrier to deep carbon subduction. Nature.

[16] Sun C, Dasgupta R (2019). Slab–mantle interaction, carbon transport, and kimberlite generation in the deep upper mantle. Earth Planet Sci. Lett..

[17] Stagno V, Ojwang DO, McCammon CA, Frost DJ (2013). The oxidation state of the mantle and the extraction of carbon from Earth’s interior. Nature.

[18] Brenker FE (2007). Carbonates from the lower part of transition zone or even the lower mantle. Earth Planet Sci. Lett..

[19] Wirth R, Kaminsky F, Matsyuk S, Schreiber A (2009). Unusual micro- and nano-inclusions in diamonds from the Juina Area, Brazil. Earth Planet Sci. Lett..

[20] Kerrick DM, Connolly JA (2001). Metamorphic devolatilization of subducted marine sediments and the transport of volatiles into the Earth’s mantle. Nature.

[21] Poli S, Franzolin E, Fumagalli P, Crottini A (2009). The transport of carbon and hydrogen in subducted oceanic crust: an experimental study to 5 GPa. Earth Planet Sci. Lett..

[22] Thomson AR, Walter MJ, Lord OT, Kohn SC (2014). Experimental determination of melting in the systems enstatite-magnesite and magnesite-calcite from 15 to 80 GPa. Am. Miner..

[23] Luth RW (2004). Experimental determination of the reaction aragonite + magnesite = dolomite at 5 to 9 GPa. Contrib. Miner. Pet..

[24] Biellmann C, Gillet P, Peyronneau J, Reynard B (1993). Experimental evidence for carbonate stability in the Earth’s lower mantle. Earth Planet Sci. Lett..

[25] Seto Y, Hamane D, Nagai T, Fujino K (2008). Fate of carbonates within oceanic plates subducted to the lower mantle, and a possible mechanism of diamond formation. Phys. Chem. Min..

[26] Oganov AR, Ono S, Ma YM, Glass CW, Garcia A (2008). Novel high-pressure structures of MgCO_3_, CaCO_3_ and CO_2_ and their role in Earth’s lower mantle. Earth Planet Sci. Lett..

[27] Pickard, C. J. & Needs, R. J. Structures and stability of calcium and magnesium carbonates at mantle pressures. *Phys. Rev. B* **91**, 104101 (2015).

[28] Yao, X., Xie, C. W., Dong, X., Oganov, A. R. & Zeng, Q. F. Novel high-pressure calcium carbonates. *Phys. Rev. B* **98**, 014108 (2018).

[29] Zhang ZG, Mao Z, Liu X, Zhang YG, Brodholt J (2018). Stability and reactions of CaCO_3_ polymorphs in the Earth’s deep mantle. J. Geophys Res.-Sol. Ea.

[30] Santos SSM, Marcondes ML, Justo JF, Assali LVC (2019). Stability of calcium and magnesium carbonates at Earth’s lower mantle thermodynamic conditions. Earth Planet Sci. Lett..

[31] Isshiki M (2004). Stability of magnesite and its high-pressure form in the lowermost mantle. Nature.

[32] Boulard E (2011). New host for carbon in the deep Earth. Proc. Natl Acad. Sci. USA.

[33] Binck, J. et al. Phase stabilities of MgCO_3_ and MgCO_3_-II studied by Raman spectroscopy, X-ray diffraction, and density functional theory calculations. *Phys. Rev. Mater.* **4**, 055001 (2020).

[34] Mao Z (2011). Dolomite III: a new candidate lower mantle carbonate. Geophy. Res. Lett..

[35] Dorfman SM (2018). Carbonate stability in the reduced lower mantle. Earth Planet Sci. Lett..

[36] Maeda F (2017). Diamond formation in the deep lower mantle: a high-pressure reaction of MgCO_3_ and SiO_2_. Sci. Rep..

[37] Lobanov SS (2017). Raman spectroscopy and X-ray diffraction of *sp*^3^ CaCO_3_ at lower mantle pressures. Phys. Rev. B.

[38] Santos SSM, Marcondes ML, Justo JF, Assali LVC (2020). Calcium carbonate at high pressures and high temperatures: a first-principles investigation. Phys. Earth Planet. Inter..

[39] Kiseeva ES, Litasov KD, Yaxley GM, Ohtani E, Kamenetsky VS (2013). Melting and phase relations of carbonated eclogite at 9-21 GPa and the petrogenesis of alkali-rich melts in the deep mantle. J. Petrol..

[40] Li XY (2018). New high-pressure phase of CaCO_3_ at the topmost lower mantle: implication for the deep-mantle carbon transportation. Geophys. Res. Lett..

[41] Drewitt JWE (2019). The fate of carbonate in oceanic crust subducted into earth’s lower mantle. Earth Planet Sci. Lett..

[42] Kakizawa S, Inoue T, Suenami H, Kikegawa T (2015). Decarbonation and melting in MgCO_3_–SiO_2_ system at high temperature and high pressure. J. Mineral. Petrol. Sci..

[43] Frost DJ, McCammon CA (2008). The redox state of Earth’s mantle. Annu. Rev. Earth Planet. Sci..

[44] Shirey SB (2013). Diamonds and the geology of mantle carbon. Carbon Earth.

[45] Stagno, V. et al. Carbon-Bearing Phases throughout Earth’s Interior. In *Deep Carbon: Past to Present,* (eds. Orcutt, B. N., Daniel, I. & Dasgupta, R.) 66–88 10.1017/9781108677950.004 (Cambridge University Press, UK 2019).

[46] Ionov DA (2019). Calcium isotopic signatures of carbonatite and silicate metasomatism, melt percolation and crustal recycling in the lithospheric mantle. Geochim Cosmochim. Acta.

[47] Wang SJ, Teng FZ, Li SG (2014). Tracing carbonate-silicate interaction during subduction using magnesium and oxygen isotopes. Nat. Commun..

[48] Nestola F (2018). CaSiO_3_ perovskite in diamond indicates the recycling of oceanic crust into the lower mantle. Nature.

[49] Chen C (2018). Calcium isotope evidence for subduction-enriched lithospheric mantle under the northern North China Craton. Geochim. Cosmochim. Acta.

[50] Kang J-T (2017). Calcium isotopic fractionation in mantle peridotites by melting and metasomatism and Ca isotope composition of the Bulk Silicate Earth. Earth Planet Sci. Lett..

[51] Amsellem E (2020). Calcium isotopic evidence for the mantle sources of carbonatites. Sci. Adv..

[52] Corgne A, Liebske C, Wood BJ, Rubie DC, Frost DJ (2005). Silicate perovskite-melt partitioning of trace elements and geochemical signature of a deep perovskitic reservoir. Geochim. Cosmochim. Acta.

[53] Howell D (2020). Deep carbon through time: Earth’s diamond record and its implications for carbon cycling and fluid speciation in the mantle. Geochim. Cosmochim. Acta.

[54] Prakapenka VB (2008). Advanced flat top laser heating system for high pressure research at GSECARS: application to the melting behavior of germanium. High. Press. Res..

[55] Dorfman SM (2020). Effects of composition and pressure on electronic states of iron in bridgmanite. Am. Miner..

[56] Hrubiak R, Sinogeikin S, Rod E, Shen G (2015). The laser micro-machining system for diamond anvil cell experiments and general precision machining applications at the high pressure collaborative access team. Rev. Sci. Instrum..

[57] Akahama Y, Kawamura H (2006). Pressure calibration of diamond anvil Raman gauge to 310GPa. J. Appl. Phys..

[58] Nomura R (2014). Low core-mantle boundary temperature inferred from the solidus of pyrolite. Science.

[59] Fiquet G (2010). Melting of peridotite to 140 gigapascals. Science.

[60] Prescher C, Prakapenka VB (2015). DIOPTAS: a program for reduction of two-dimensional X-ray diffraction data and data exploration. High. Press. Res..

