# Supplementary material for: Reversal of carbonate-silicate cation exchange in cold slabs in Earth’s lower mantle
Source: Nat Commun. 2021 Mar 17;12:1712. doi: 10.1038/s41467-021-21761-9 (PMC7969735; doi:10.1038/s41467-021-21761-9)
Supplement: Supplementary file 1 — Supplementary Information [file 41467_2021_21761_MOESM1_ESM.pdf]

Supplementary information for

Reversal of carbonate-silicate cation exchange in cold slabs in Earth's lower mantle

Authors: Mingda Lv[1,*], Susannah M. Dorfman[1,*], James Badro[2], Stephan Borensztajn[2], Eran Greenberg[3], Vitali B. Prakapenka[3]

Affiliations:

[1] Department of Earth and Environmental Sciences, Michigan State University, Michigan 48824, USA

[2] Université de Paris, Institut de physique du globe de Paris, CNRS, Paris 75005, France

[3] Center for Advanced Radiation Sources, University of Chicago, IL60637, USA

* Correspondence to: M. Lv (lyumingd@msu.edu) and S.M. Dorfman (dorfman3@msu.edu)

Supplementary information include:

Supplementary Note 1

Supplementary Figures 1-13

Supplementary Tables 1-2

Supplementary References (11)

Supplementary Note 1

To assess whether observations of Ca and Mg isotopes in mantle silicates or diamond inclusions can be used to detect the presence of carbonates in the deep mantle, we estimate the potential effects of carbonate-silicate cation exchange on isotope signatures based on available constraints and mass balance.

Subducting carbonates carry low-$\delta^{44/40}Ca$ (reported relative to NIST SRM 915a standard, $\delta^{44/40}Ca = [(^{44}Ca/^{40}Ca)_{sample}/(^{44}Ca/^{40}Ca)_{standard} - 1]\times1,000$) and low-$\delta^{26}Mg$ (relative to the Dead Sea metal Mg standard (DSM-3, Galy et al., 2003), $\delta^{26}Mg = [(^{26}Mg/^{24}Mg)_{sample}/(^{26}Mg/^{24}Mg)_{standard} - 1]\times1,000$) signatures, while reported mantle ratios are heavier (Fantle & Tipper, 2014; Kang et al., 2017; Teng et al., 2010; Wombacher et al., 2011)(Supplementary Table 2). Based on both our observations and previous experimental studies (Biellmann et al., 1993; Seto et al., 2008), $CaCO_3$ is unstable relative to $MgCO_3$ in the shallow lower mantle due to the reaction CaC-to-MgC, so the light Ca isotopes brought by $CaCO_3$ may be transferred to $CaSiO_3$ in the slab and surrounding ambient shallow lower mantle. Conversely, our experiments obtain the new result that in the deep lower mantle, $MgCO_3$ is unstable relative to $CaCO_3$ due to the reaction MgC-to-CaC, so the light Mg isotopes brought by $MgCO_3$ may be transferred to the surrounding ambient deep lower mantle in $MgSiO_3$, and deep mantle $CaCO_3$ would form with the Ca isotope signature of the mantle silicate. These reactions, their stable isotope equilibrium fractionation factors, and the masses of carbonate and silicate that reach equilibrium may moderate isotope ratios in cold carbonated subducting slabs and their surroundings.

The reaction CaC-to-MgC affects $\delta^{44/40}Ca$ of mantle and $\delta^{26}Mg$ of carbonate:

$$(f_{Ca\_pyro} + k \cdot f_{Ca\_carb}) \cdot \delta^{44/40}Ca_{pyro} = f_{Ca\_pyro} \cdot \delta^{44/40}Ca_{pyro}^{i} + k \cdot f_{Ca\_carb} \cdot \delta^{44/40}Ca_{carb}^{i} \quad (1)$$

$$(f_{Mg\_carb} + k \cdot f_{Ca\_carb}) \cdot \delta^{26}Mg_{carb} = f_{Mg\_carb} \cdot \delta^{26}Mg_{carb}^{i} + k \cdot f_{Ca\_carb} \cdot \delta^{26}Mg_{pyro}^{i} \quad (2)$$

and the reaction MgC-to-CaC affects δ^{26}Mg of mantle and $\delta^{44/40}$Ca of carbonate:

$$(f_{Mg\_pyro} + k \cdot f_{Mg\_carb}) \cdot \delta^{26}Mg_{pyro} = f_{Mg\_pyro} \cdot \delta^{26}Mg_{pyro}^{i} + k \cdot f_{Mg\_carb} \cdot \delta^{26}Mg_{carb}^{i} \quad (3)$$

$$(f_{Ca\_carb} + k \cdot f_{Mg\_carb}) \cdot \delta^{44/40}Ca_{carb} = f_{Ca\_carb} \cdot \delta^{44/40}Ca_{carb}^{i} + k \cdot f_{Mg\_carb} \cdot \delta^{44/40}Ca_{pyro}^{i} \quad (4)$$

where f_{Ca_pyro}, f_{Ca_carb}, f_{Mg_pyro}, f_{Mg_carb} represent the mole fraction of Ca and Mg contributed by pyrolitic mantle and carbonate, respectively. i represents the initial status before the reaction. *k* is reaction rate ranging from 0 to 1, representing the mole fraction of carbonate that undergoes cation exchange reaction with silicate in pyrolitic mantle. In this model, we fixed the weight ratio of carbonate and surrounding pyrolitic mantle to 1/10 based on previous work applied to upper mantle conditions (Wang et al., 2014). For a subducting slab in the lower mantle, this ratio represents a generous upper bound on the amount of carbonate available to react with silicates. We varied the composition of subducted carbonate by changing the n in $(Mg_nCa_{n-1})CO_3$ from 0 to 1. Parameters used in the calculation are listed in Table 1, and the calculated results are plotted in Supplementary Fig. 12.

We assume equilibrium isotope fractionation between carbonates and mantle silicates, occurring after the cation exchange reaction, i.e., carbonates are well mixed and equilibrium with surrounding mantle. In this case, $\delta^{44/40}$Ca and δ^{26}Mg of this carbonated pyrolite are governed by the following equations according to Wang et al. (2014), respectively:

$$(f_{Ca\_pyro} + k \cdot f_{Ca\_carb})\ \delta^{44/40}Ca_{pyro}^{f} = (f_{Ca\_pyro} + k \cdot f_{Ca\_carb}) \cdot \delta^{44/40}Ca_{pyro} +$$
$$(\delta^{44/40}Ca_{carb}^{i} + \Delta^{44/40}Ca_{pyro\text{-}carb}) \cdot (f_{Ca\_carb} - k \cdot f_{Ca\_carb}) \quad (5)$$

$$(f_{Mg\_pyro} + k \cdot f_{Mg\_carb}) \cdot \delta^{26}Mg_{pyro}^{f} = (f_{Mg\_pyro} - k \cdot f_{Ca\_carb}) \cdot \delta^{26}Mg_{pyro}^{i} +$$
$$(\delta^{26}Mg_{carb} + \Delta^{26}Mg_{\ pyro\text{-}carb}) \cdot (f_{Mg\_carb} + k \cdot f_{Ca\_carb}) \quad (6)$$

similarly, after the reaction MgC-to-CaC, $\delta^{44/40}$Ca and δ^{26}Mg of mantle are governed by the following equations, respectively:

$$(f_{Mg\_pyro} + k \cdot f_{Mg\_carb}) \cdot \delta^{26}Mg_{pyro}{}^{f} = (f_{Mg\_pyro} + k \cdot f_{Mg\_carb}) \cdot \delta^{26}Mg_{pyro} + (\delta^{26}Mg_{carb}{}^{i} + \Delta^{26}Mg_{pyro\text{-}carb}) \cdot (f_{Mg\_carb} - k \cdot f_{Mg\_carb}) \quad (7)$$

$$(f_{Ca\_pyro} + k \cdot f_{Ca\_carb}) \cdot \delta^{44/40}Ca_{pyro}{}^{f} = (f_{Ca\_pyro} - k \cdot f_{Mg\_carb}) \cdot \delta^{44/40}Ca_{pyro}{}^{i} + (\delta^{44/40}Ca_{carb} + \Delta^{44/40}Ca_{pyro\text{-}carb}) \cdot (f_{Ca\_carb} + k \cdot f_{Mg\_carb}) \quad (8)$$

where f represents the final status after the isotope fractionation. $\Delta^{44/40}Ca_{pyro\text{-}carb}$ and $\Delta^{26}Mg_{pyro\text{-}carb}$ represent equilibrium isotope fractionation between silicate and carbonate. Values for $\Delta^{44/40}Ca_{pyro\text{-}carb}$ and $\Delta^{26}Mg_{pyro\text{-}carb}$ listed in Supplementary Table 2 represent constraints measured at ~Earth surface conditions. Fractionation may be expected to depend on pressure and temperature as well as the high-pressure polymorphism in the carbonate and silicate systems. However, due to the lack of available measurements at deep mantle conditions, we assume fractionation values remain constant throughout the mantle. The calculated results are plotted in Supplementary Fig. 13.

To use isotope fractionation to identify carbonates at depth, isotope ratios in subducted carbonate cation exchange scenarios must be significantly different from the scenario where no subducted carbonate reaches the deep mantle. If no carbonate reaches the lower mantle in subducted slabs, lower-mantle-derived silicates will have heavy $\delta^{44/40}Ca$ and $\delta^{26}Mg$, and there would be no lower-mantle-derived carbonate. If some carbonate reaches the shallow lower mantle in subducted slabs, lower-mantle-derived silicates from this region may have light $\delta^{44/40}Ca$ due to reaction of persistent metastable $CaCO_3$ with $MgSiO_3$ to produce $CaSiO_3$ (reaction CaC-MgC). The mass balance calculation indicates that a generous upper bound on the masses involved could locally enrich mantle $CaSiO_3$ in light $\delta^{44/40}Ca$ (Supplementary Fig. 12a). There would be no effect on $\delta^{26}Mg$ of the silicates in the mantle, the CaC-MgC reaction would make $\delta^{26}Mg$ in the carbonate relatively heavy (Supplementary Fig. 12a) and would no longer appear to have a subduction source. If some carbonate continues to the deeper lower mantle where the reaction MgC-CaC

becomes favorable, the much greater abundance of Mg in mantle silicates means that δ^{26}Mg of silicates would not be significantly impacted by the breakdown of $MgCO_3$ (Supplementary Fig. 12b). However, the $\delta^{44/40}$Ca in $CaCO_3$ produced by this reaction would reflect the heavy mantle source (Supplementary Fig. 12b) and could be distinguished by $CaCO_3$ with a surface origin. The exchange reactions potentially overwrite the isotope signals in subducted carbonates with heavy isotopes, and could not significantly affect mantle silicate isotope ratios in the deep lower mantle. Mg and Ca isotope composition of carbonated pyrolite after the equilibrium isotopic fractionation between carbonate and surrounding pyrolitic mantle depends on the mole ratio of Mg and Ca of the subducted carbonates, but less strongly depends relies on the reaction completion (Supplementary Fig. 13). In summary, carbonate-silicate cation exchange reactions only produce potentially observable heterogeneity in silicate cation isotopes relative to the null case in the CaC-MgC regime, and only in $\delta^{44/40}$Ca.

$CaCO_3$ formed by a cation exchange reaction between $MgCO_3$ and $CaSiO_3$ in the lower mantle can be expected to have a different isotope signature relative to subducted $CaCO_3$ formed at Earth's surface. $CaCO_3$ formed in the deep lower mantle would contain Ca isotopes that sample the "ambient mantle" source rather than a subducted carbonate source. This could provide a test for shallow vs. ultradeep origin of carbonate inclusions. This is the motivation for future systematic study of the isotope signatures of diamond inclusions.

Supplementary Figures

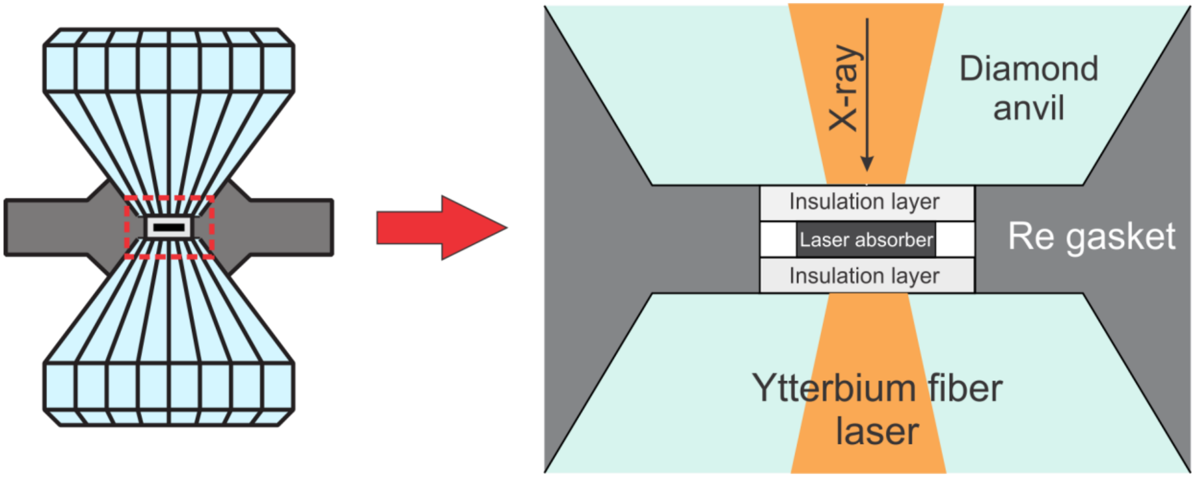

Supplementary Fig. 1. Schematic diagram of the laser-heated diamond-anvil cell (LHDAC) and sample loading design. The insulation layer (light gray region) and the laser absorber (black region) for different experimental runs are summarized in Supplementary Table 1.

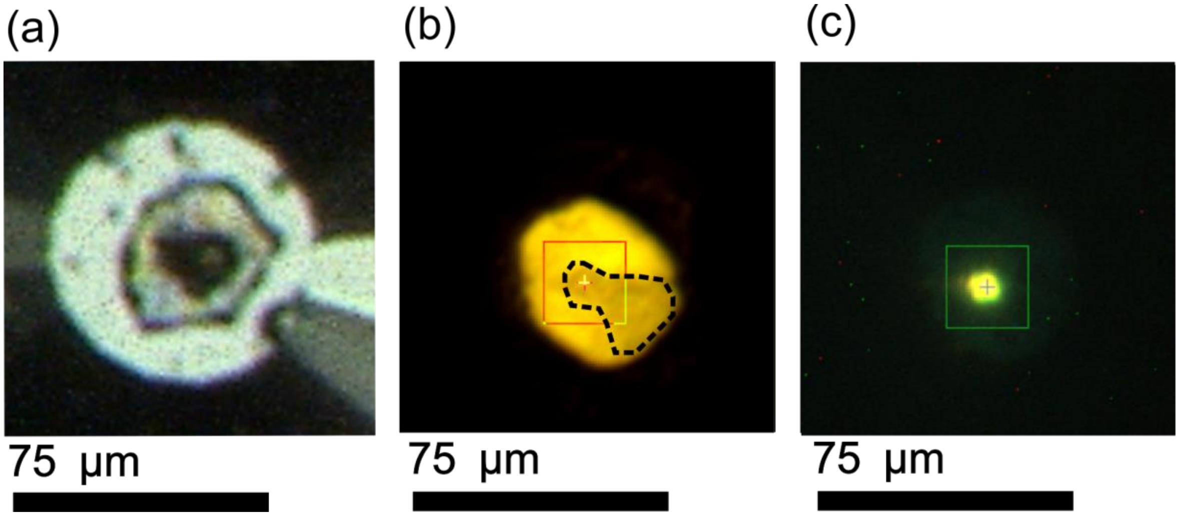

Supplementary Fig. 2. Microscope images of loaded sample for run #9. We loaded the Fe-bearing sample on top of the thermal insulation layer on the piston side of DAC (75/300 beveled anvil), then we loaded another insulation layer on the cylinder side of DAC together with Re gasket before we close and compress the DAC to the target pressure. (a) Samples are loaded at ambient conditions on the piston side before closing the cell. (b) Samples are compressed to target pressure before heating, and the dashed circle indicates the dark Fe-bearing sample. (c) The heating spot on the loaded sample during laser heating.

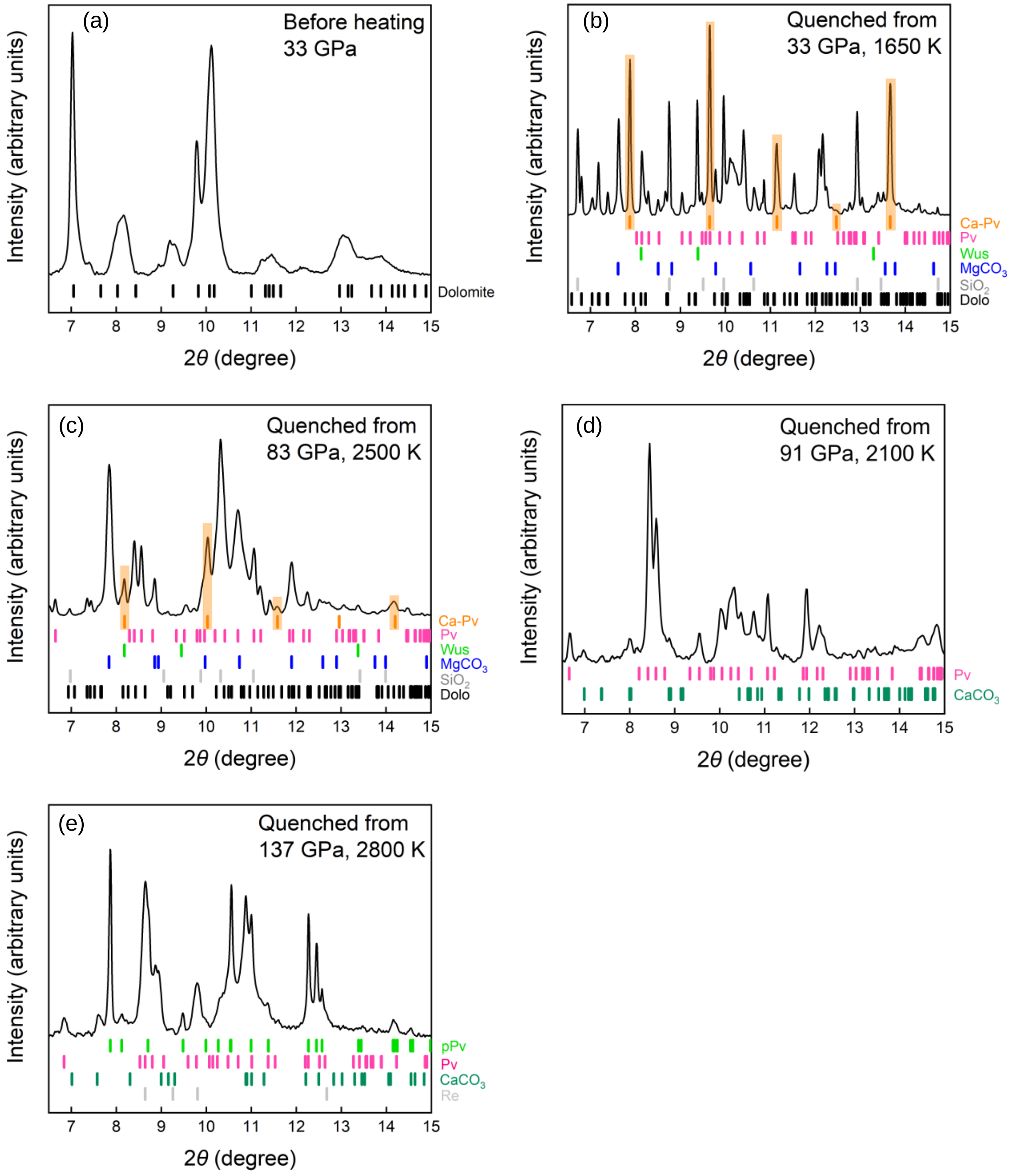

Supplementary Fig. 3. X-ray diffraction patterns obtained from the starting material of CaC-to-MgC before heating (a) and products quenched from various *P*-*T* conditions: (b) run #1, (c) run #4, (d) run #5, and (e) run #7, and phase identifications are indicated by small ticks at the bottom. The wavelength of the incident X-ray beam was 0.3344 Å.

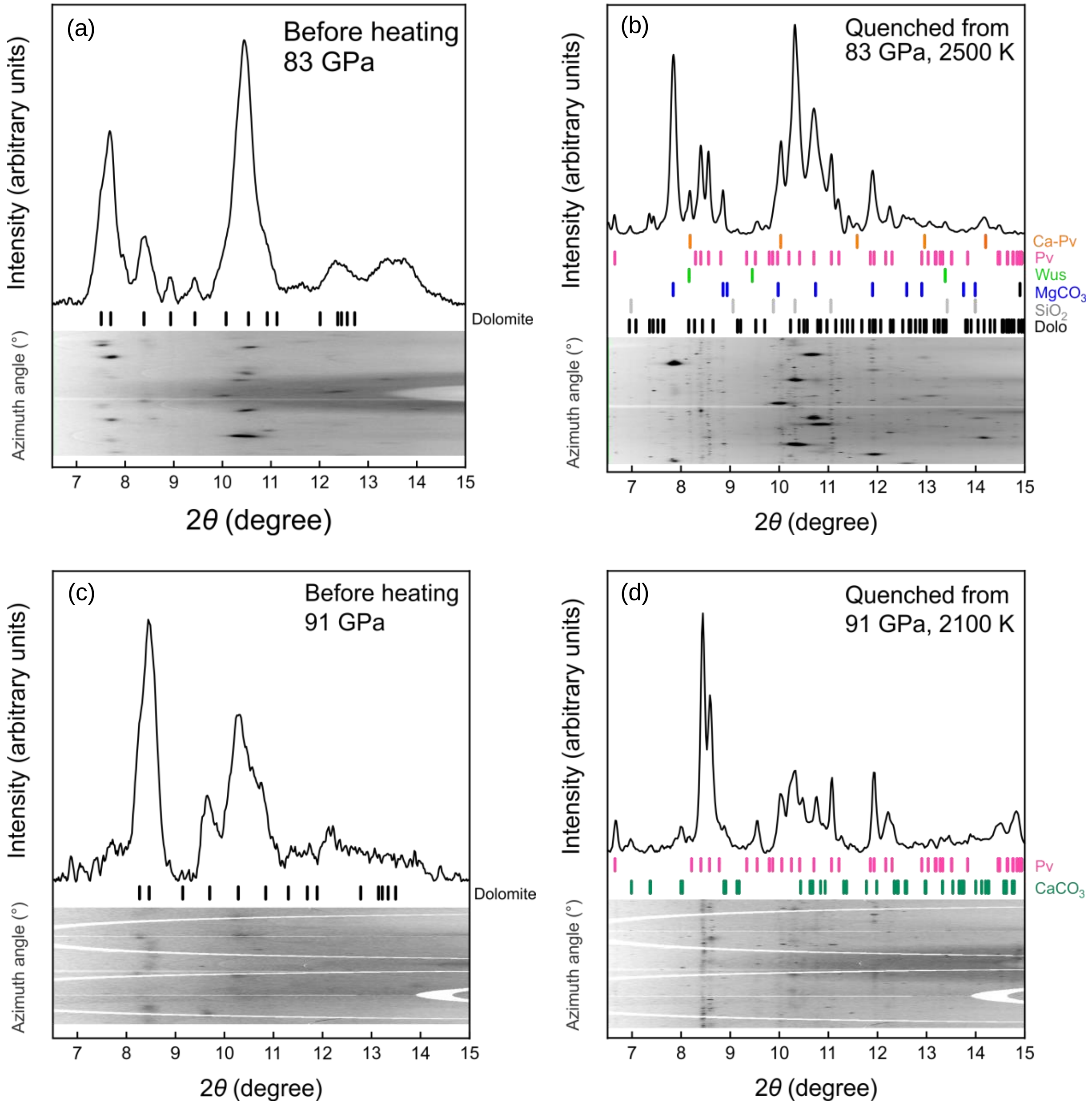

Supplementary Fig. 4. Representative unrolled X-ray diffraction images (lower panel) corresponding to X-ray diffraction patterns (upper panel) obtained from the starting materials of CaC-to-MgC before heating and the products quenched from various *P*-*T* conditions: (a-b) run #4, (c-d) run #5. Large spots in 2D diffraction patterns in (a) and (b) are from untransformed dolomite starting material. The wavelength of the incident X-ray beam was 0.3344 Å.

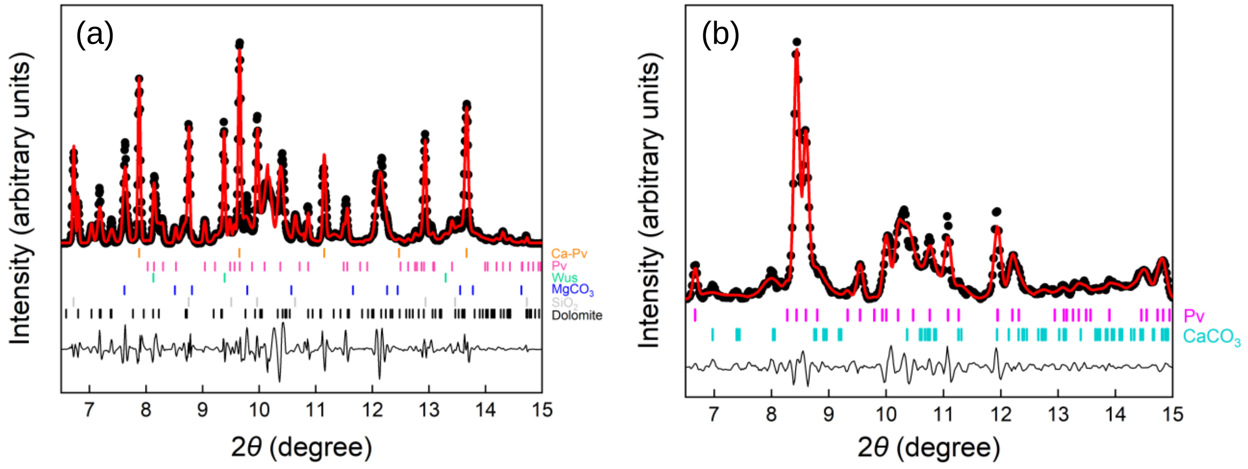

Supplementary Fig. 5. Representative full-profile fitting for XRD of (a) run #1 and (b) run #5. Le Bail refinements (red curves) of observed XRD data (black dots) were carried out after background subtraction, demonstrating all the identified phases (vertical ticks below patterns) can account for the peaks and intensities of XRD patterns. Black curves are fitting residues. The wavelength of the incident X-ray beam was 0.3344 Å.

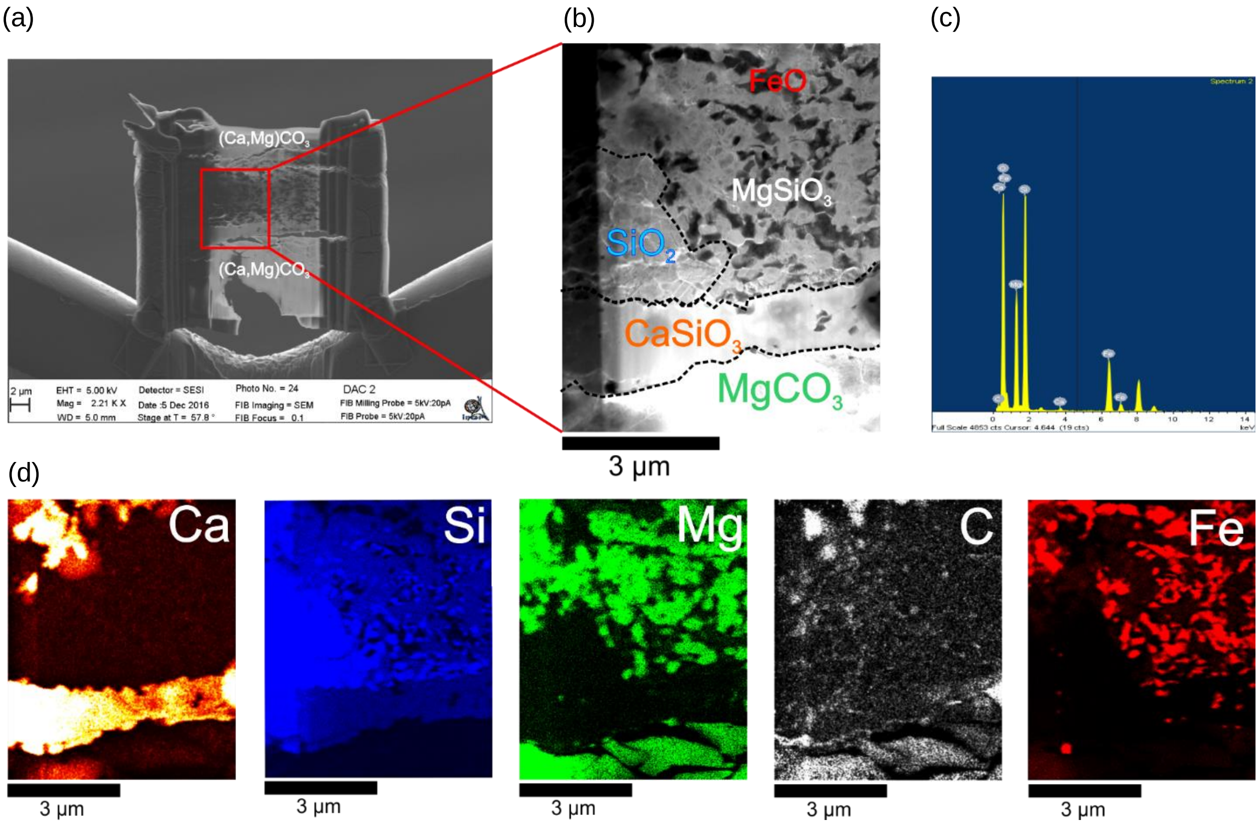

Supplementary Fig. 6. Ex-situ analysis of sample quenched from 33 GPa and 1650 K heated for 15 min (run #1) demonstrates CaC-to-MgC. (a) SEM-BSE image obtained during FIB milling. Sample was prepared as a $(Mg,Fe)SiO_3$ layer sandwiched by two $(Ca,Mg)CO_3$ layers; (b) dark-field STEM image reveals $CaSiO_3$ and $MgCO_3$, as well as SiO_2 and FeO, formed by reaction between $(Mg,Fe)SiO_3$ and $(Ca,Mg)CO_3$ layers; (c) EDX spectrum and corresponding (d) chemical maps for calcium, silicon, magnesium, carbon, and iron.

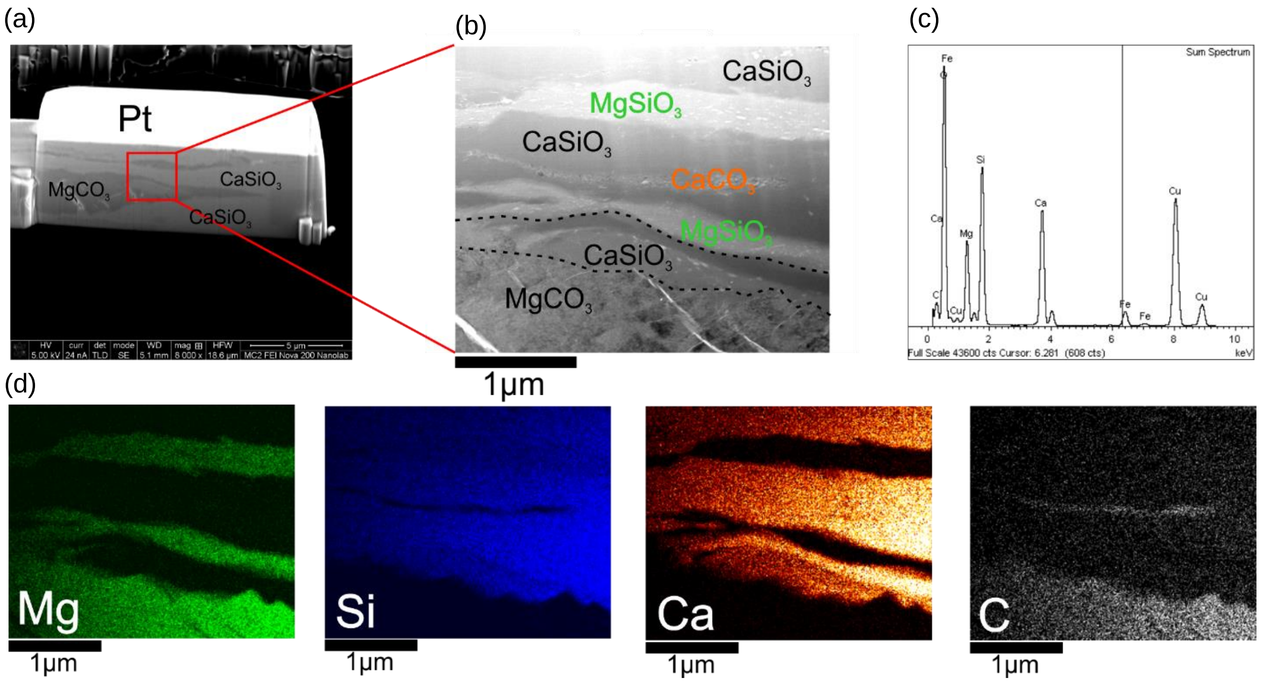

Supplementary Fig. 7. Ex-situ analysis of sample quenched from 88 GPa and 1800 K heated for 150 min (run #9) demonstrates MgC-to-CaC. (a) SEM-BSE image obtained during FIB milling. Sample was prepared as an $(Mg,Fe)CO_3$ layer sandwiched by two $CaSiO_3$ layers; (b) dark-field STEM image reveals $(Mg,Fe)SiO_3$ and $CaCO_3$ formed by reaction of $(Mg,Fe)CO_3$ and $CaSiO_3$ layers; (c) STEM-EDX spectrum and corresponding (d) chemical maps for magnesium, silicon, calcium, and carbon.

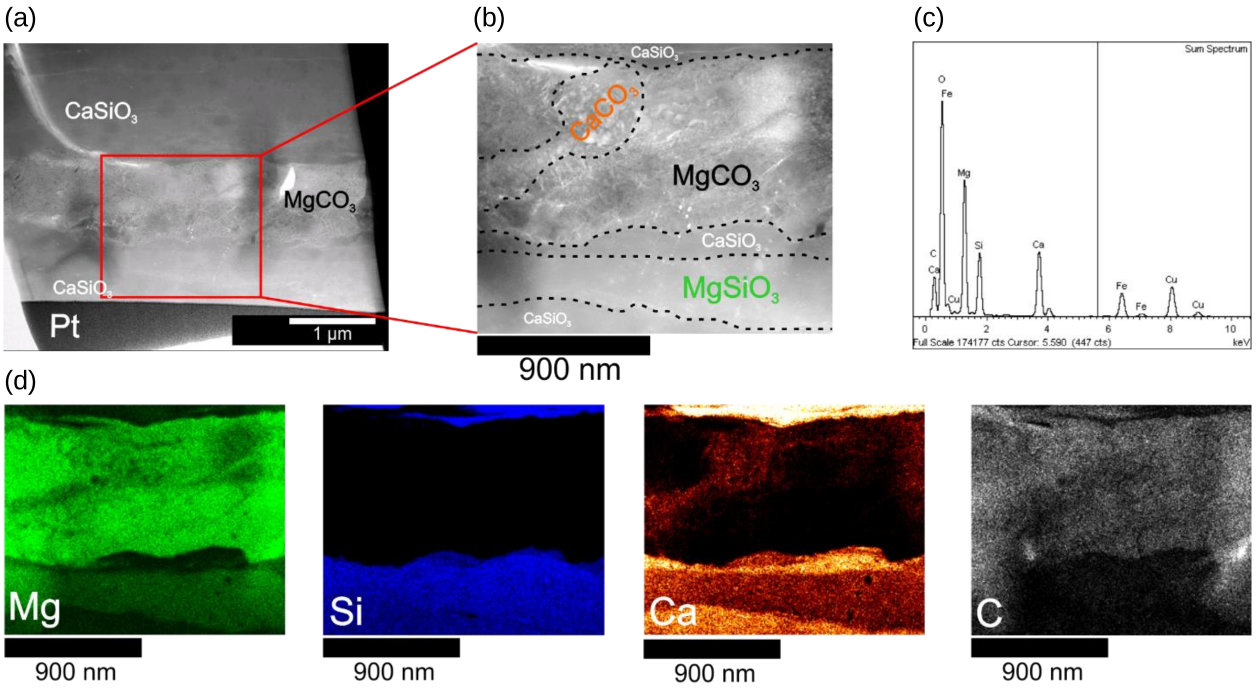

Supplementary Fig. 8. Ex-situ analysis of sample quenched from 133 GPa and 2000 K heated for 400 min (run #10) demonstrates MgC-to-CaC. (a) SEM-BSE image obtained during FIB milling. Sample was prepared as a $(Mg,Fe)CO_3$ layer sandwiched by two $CaSiO_3$ layers; (b) dark-field STEM image reveals $(Mg,Fe)SiO_3$ and $CaCO_3$ formed by reaction of $(Mg,Fe)CO_3$ and $CaSiO_3$ layers; (c) EDX spectrum and corresponding (d) chemical maps for magnesium, silicon, calcium, and carbon.

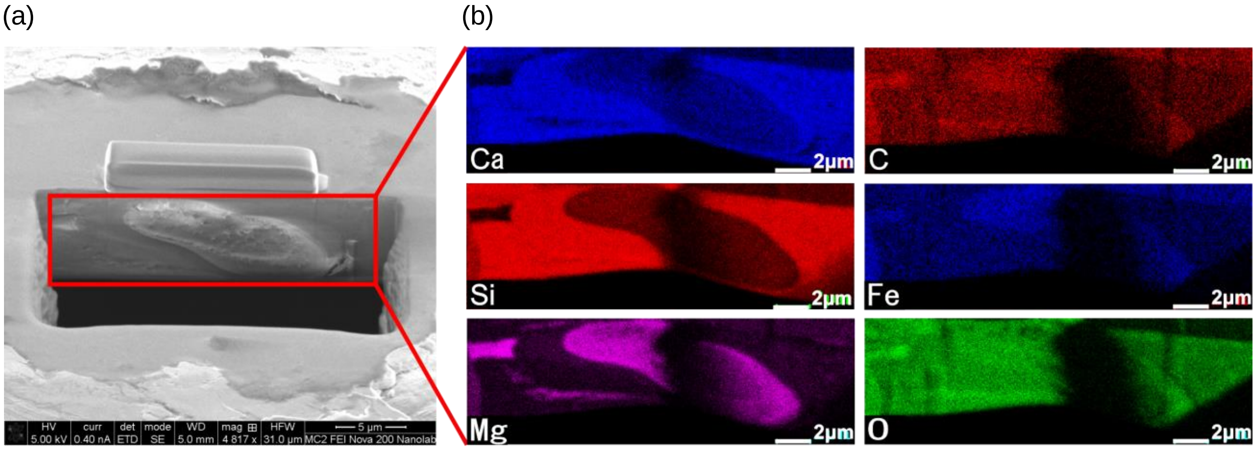

Supplementary Fig. 9. Ex-situ analysis of sample quenched from 35 GPa and 1900 K heated for 20 min (run #8) demonstrates MgC-to-CaC. (a) SEM-BSE image obtained during FIB milling. Sample was prepared as a $(Mg,Fe)CO_3$ layer sandwiched by two $CaSiO_3$ layers; (b) STEM-EDX chemical maps for calcium, silicon, magnesium, carbon, iron and oxygen.

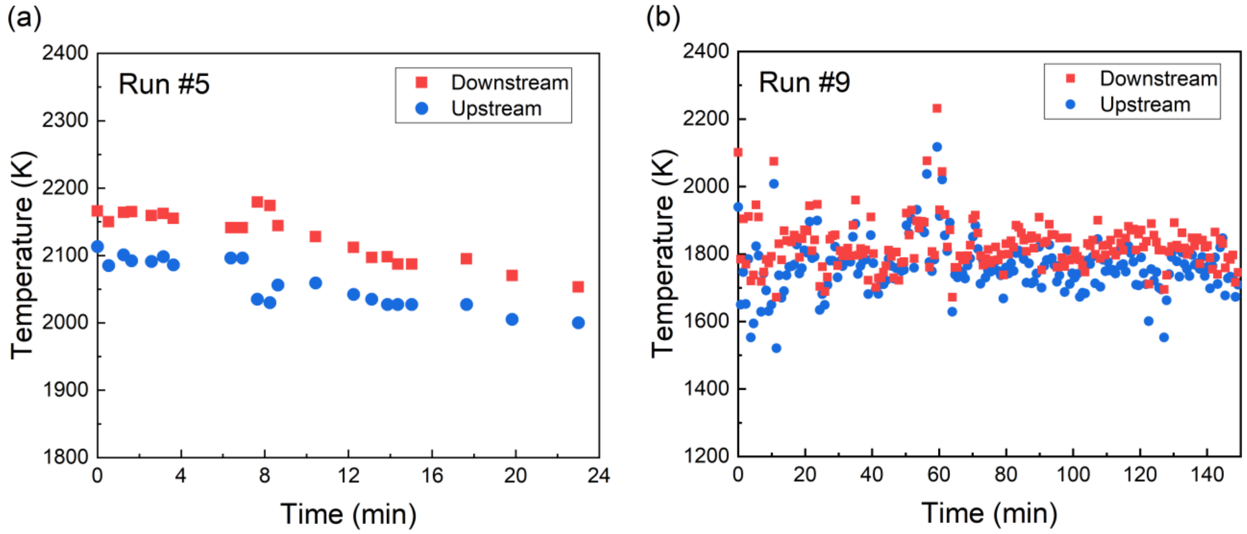

Supplementary Fig. 10. Typical temperature measurements of downstream (red squares) and upstream (blue circles) over heating duration of (a) run #5 for CaC-to-MgC and (b) run #9 for MgC-to-CaC, respectively.

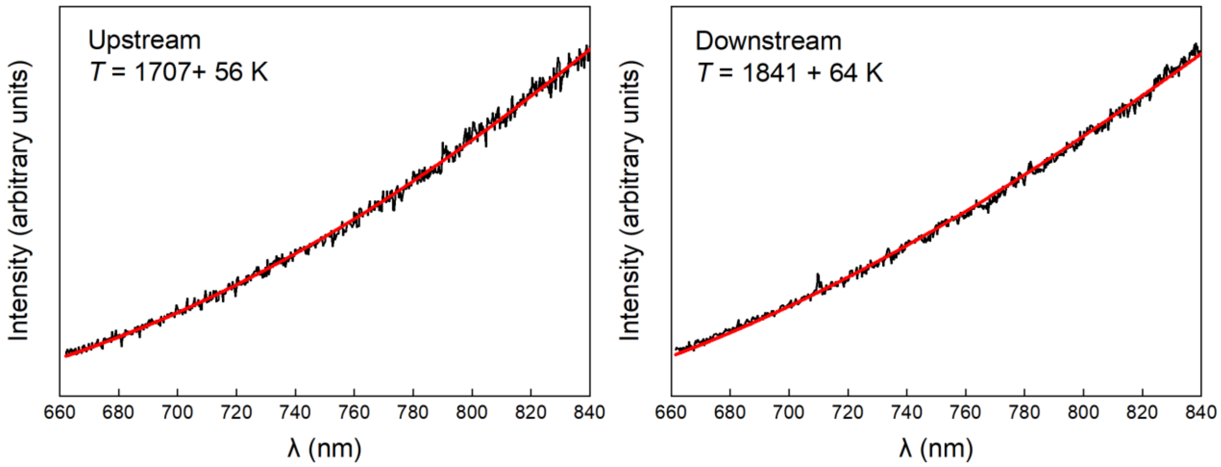

Supplementary Fig. 11. Representative temperature measurements and fitting profiles of upstream and downstream for run #9. Temperatures of the heated samples were determined by fitting the measured thermal radiation spectra using the Planck radiation function under the graybody approximation.

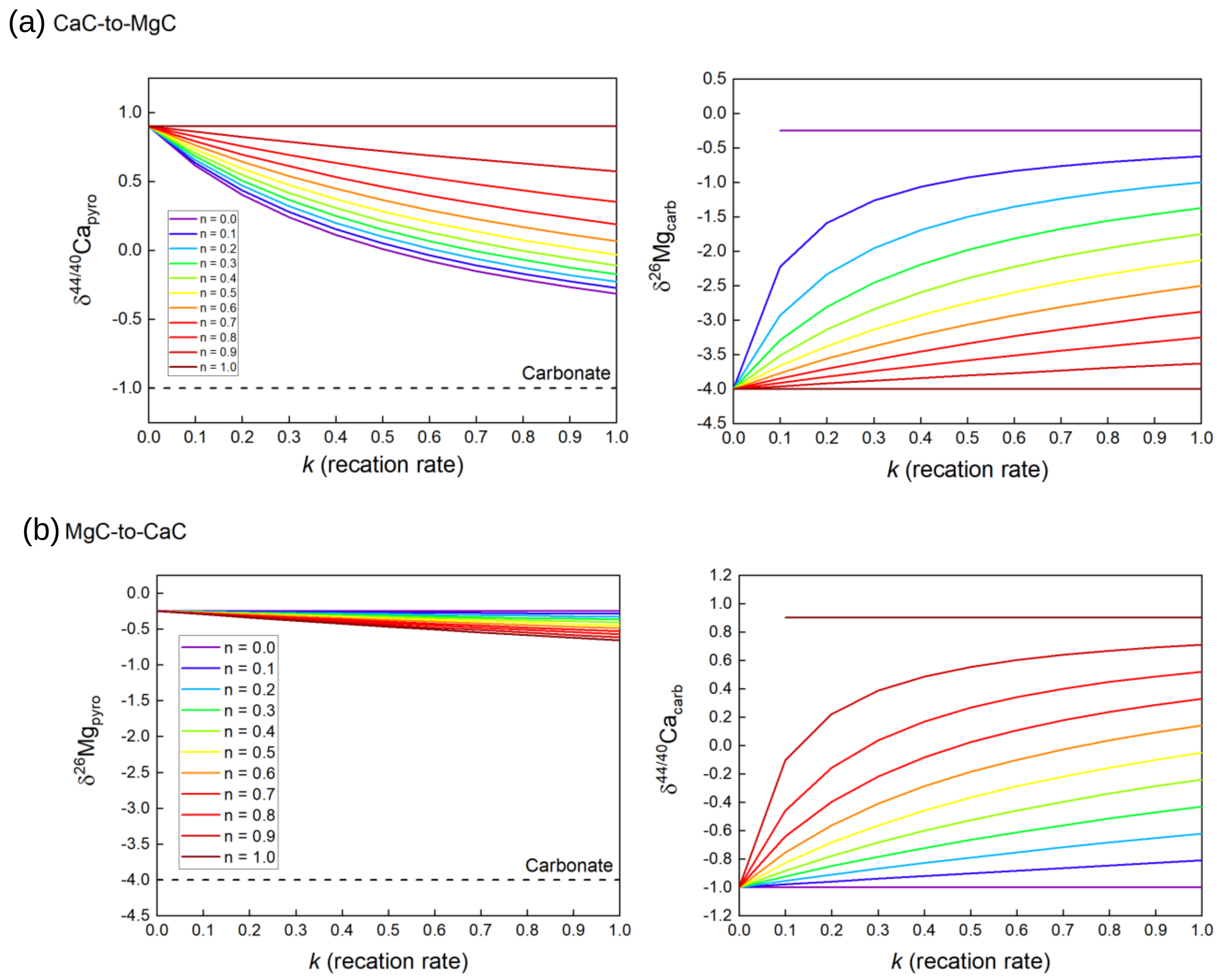

Supplementary Fig. 12. Calculated isotopic composition versus reaction rate after the reaction (a) CaC-to-MgC and (b) MgC-to-CaC. n represents the mole fraction of Mg in $(Mg_nCa_{n-1})CO_3$. Black dashed line represents the average $\delta^{44/40}$Ca and δ^{26}Mg values in carbonates, respectively.

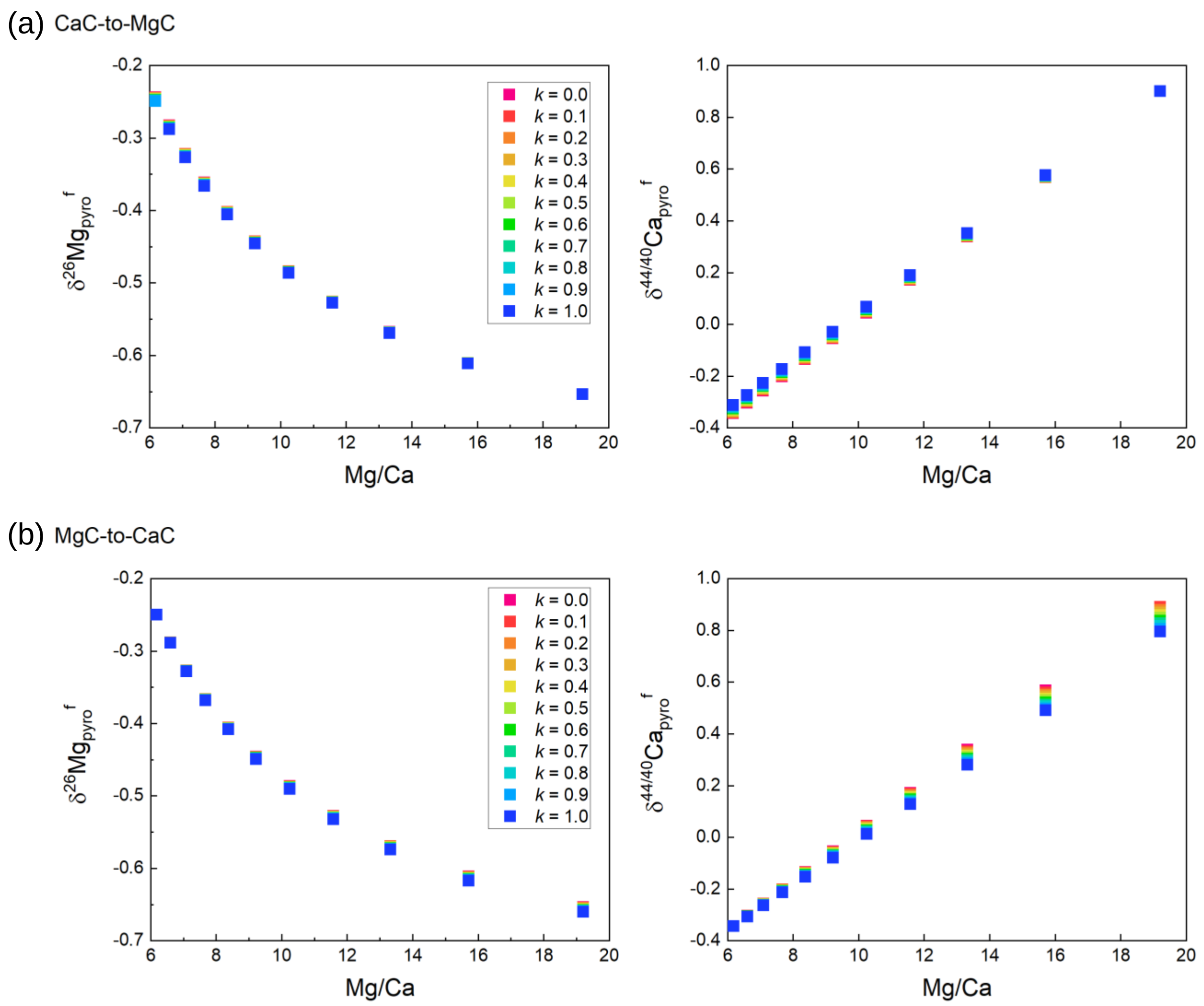

Supplementary Fig. 13. Calculated isotopic composition of carbonated pyrolite after isotopic fractionation between carbonates and silicates for the reaction (a) CaC-to-MgC and (b) MgC-to-CaC. The horizontal axis represents the mole ratio of Mg/Ca in the carbonated pyrolite. *k* represents reaction rate.

Supplementary Tables

Supplementary Table 1. Starting materials, experimental conditions, and run products for all experiments. Starting materials for experiments were loaded in sandwich configuration, with laser absorber layer between two thermal insulation layers. Pressures determined from Raman shift of the singlet peak of the diamond anvil at the culet surface (Akahama & Kawamura, 2006) with 2σ uncertainties. The temperature reported is the temporal average of recorded temperatures over the heating duration rounded to the nearest 50 K. Temperature fluctuations over this time scale were less than the specified uncertainty, which is derived from a standard deviation of temperature measurements from both sides of the laser-heated sample (typically ±100 K below 2000 K and ±150 K above 2000 K).

Run #	Insulation	Laser absorber	*P* (GPa)	*T* (K)	Heating duration (min)	Phases observed after heating
1	$(Mg,Ca)CO_3$	$(Mg,Fe)SiO_3$	33(2)[a]	1650(100)	15	$CaSiO_3 + SiO_2 + FeO + MgSiO_3 + MgCO_3$
2	$(Mg,Ca)CO_3$	$(Mg,Fe)SiO_3$	45(2)	1600(100)	20	$CaSiO_3 + MgSiO_3 + MgCO_3$
3	$(Mg,Ca)CO_3$	$(Mg,Fe)SiO_3$	66(3)	1900(100)	11	$CaSiO_3 + MgSiO_3 + MgCO_3$
4	$(Mg,Ca)CO_3$	$(Mg,Fe)SiO_3$	83(4)	2500(150)	20	$CaSiO_3 + MgSiO_3 + MgCO_3$
5	$(Mg,Ca)CO_3$	$(Mg,Fe)SiO_3$	91(5)	2100(150)	24	$MgSiO_3 + CaCO_3$
6	$(Mg,Ca)CO_3$	$(Mg,Fe)SiO_3$	106(5)	2000(150)	10	$MgSiO_3 + CaCO_3$
7	$(Mg,Ca)CO_3$	$(Mg,Fe)SiO_3$	137(7)	2800(150)	15	$MgSiO_3 + CaCO_3$
8	$CaSiO_3$	$(Mg,Fe)CO_3$	35(2)	1900(100)	20	$CaSiO_3 + MgCO_3$
9	$CaSiO_3$	$(Mg,Fe)CO_3$	88(4)	1800(100)	150	$MgSiO_3 + CaCO_3 + CaSiO_3 + MgCO_3$
10	$CaSiO_3$	$(Mg,Fe)CO_3$	133(7)	2000(150)	400	$MgSiO_3 + CaCO_3 + CaSiO_3 + MgCO_3$

[a] Numbers in parenthesis are uncertainties on the last digits.

Supplementary Table 2. Parameters for isotopic mass balance calculations (see Supplementary Note 1 for details).

Parameter	Value	Reference
$\delta^{44/40}Ca_{pyro}{}^{i}$	0.9 ‰	Kang et al. (2017)
$\delta^{44/40}Ca_{carb}{}^{i}$	-1.0 ‰	Fantle and Tipper (2014)
$\delta^{26}Mg_{pyro}{}^{i}$	-0.25 ‰	Teng et al. (2010)
$\delta^{26}Mg_{carb}{}^{i}$	-4.0 ‰	Wombacher et al. (2011)
CaO abundance in pyrolite	3.17 %	Workman and Hart (2005)
MgO abundance in pyrolite	38.73 %	Workman and Hart (2005)
$\Delta^{44/40}Ca_{pyro\text{-}carb}$	-0.05 ‰	Amsellem et al. (2020)
$\Delta^{26}Mg_{pyro\text{-}carb}$	0.06 ‰	Macris et al. (2013)

Supplementary References

Akahama, Y., & Kawamura, H. (2006). Pressure calibration of diamond anvil Raman gauge to 310GPa. *Journal of Applied Physics, 100*(4), 043516. http://10.1063/1.2335683

Amsellem, E., Moynier, F., Bertrand, H., Bouyon, A., Mata, J., Tappe, S., & Day, J. M. D. (2020). Calcium isotopic evidence for the mantle sources of carbonatites. *Sci Adv, 6*(23), eaba3269. http://10.1126/sciadv.aba3269

Biellmann, C., Gillet, P., Peyronneau, J., & Reynard, B. (1993). Experimental evidence for carbonate stability in the Earth's lower mantle. *Earth and Planetary Science Letters, 118*(1-4), 31-41.

Fantle, M. S., & Tipper, E. T. (2014). Calcium isotopes in the global biogeochemical Ca cycle: implications for development of a Ca isotope proxy. *Earth-Science Reviews, 129*, 148-177.

Kang, J.-T., Ionov, D. A., Liu, F., Zhang, C.-L., Golovin, A. V., Qin, L.-P., et al. (2017). Calcium isotopic fractionation in mantle peridotites by melting and metasomatism and Ca isotope composition of the Bulk Silicate Earth. *Earth and Planetary Science Letters, 474*, 128-137.

Macris, C. A., Young, E. D., & Manning, C. E. (2013). Experimental determination of equilibrium magnesium isotope fractionation between spinel, forsterite, and magnesite from 600 to 800 C. *Geochimica et Cosmochimica Acta, 118*, 18-32.

Seto, Y., Hamane, D., Nagai, T., & Fujino, K. (2008). Fate of carbonates within oceanic plates subducted to the lower mantle, and a possible mechanism of diamond formation. *Physics and Chemistry of Minerals, 35*(4), 223-229. http://10.1007/s00269-008-0215-9

Teng, F.-Z., Li, W.-Y., Ke, S., Marty, B., Dauphas, N., Huang, S., et al. (2010). Magnesium isotopic composition of the Earth and chondrites. *Geochimica et Cosmochimica Acta, 74*(14), 4150-4166.

Wang, S. J., Teng, F. Z., & Li, S. G. (2014). Tracing carbonate-silicate interaction during subduction using magnesium and oxygen isotopes. *Nat Commun, 5*, 5328. Article. http://10.1038/ncomms6328

Wombacher, F., Eisenhauer, A., Böhm, F., Gussone, N., Regenberg, M., Dullo, W.-C., & Rüggeberg, A. (2011). Magnesium stable isotope fractionation in marine biogenic calcite and aragonite. *Geochimica et Cosmochimica Acta, 75*(19), 5797-5818.

Workman, R. K., & Hart, S. R. (2005). Major and trace element composition of the depleted MORB mantle (DMM). *Earth and Planetary Science Letters, 231*(1-2), 53-72. http://10.1016/j.epsl.2004.12.005
